# CorNet: Assigning function to networks of co-evolving residues by automated literature mining

**DOI:** 10.1371/journal.pone.0176427

**Published:** 2017-05-18

**Authors:** Tom van den Bergh, Giorgio Tamo, Alberto Nobili, Yifeng Tao, Tianwei Tan, Uwe T. Bornscheuer, Remko K. P. Kuipers, Bas Vroling, René M. de Jong, Kalyanasundaram Subramanian, Peter J. Schaap, Tom Desmet, Bernd Nidetzky, Gert Vriend, Henk-Jan Joosten

**Affiliations:** 1 Bio-Prodict, Nijmegen, The Netherlands; 2 Laboratory of Systems and Synthetic Biology, Wageningen University, Wageningen, The Netherlands; 3 Institute of Biochemistry, Department of Biotechnology & Enzyme Catalysis, Greifswald University, Greifswald, Germany; 4 Beijing Key Lab of Bioprocess, Beijing University of Chemical Technology, Chaoyang, Beijing, China; 5 DSM Biotechnology Center, Delft, The Netherlands; 6 Centre for Industrial Biotechnology and Biocatalysis, Ghent University, Ghent, Belgium; 7 Institute of Biotechnology and Biochemical Engineering, Graz University of Technology, Graz, Austria; 8 CMBI, Radboudumc, Nijmegen, The Netherlands; Indian Institute of Science, INDIA

## Abstract

CorNet is a web-based tool for the analysis of co-evolving residue positions in protein super-family sequence alignments. CorNet projects external information such as mutation data extracted from literature on interactively displayed groups of co-evolving residue positions to shed light on the functions associated with these groups and the residues in them. We used CorNet to analyse six enzyme super-families and found that groups of strongly co-evolving residues tend to consist of residues involved in a same function such as activity, specificity, co-factor binding, or enantioselectivity. This finding allows to assign a function to residues for which no data is available yet in the literature. A mutant library was designed to mutate residues observed in a group of co-evolving residues predicted to be involved in enantioselectivity, but for which no literature data is available yet. The resulting set of mutations indeed showed many instances of increased enantioselectivity.

## Introduction

The enormous progress in sequencing technology has increased the number of available sequences to hundreds of millions. For instance, the metagenome sequencing of just the biological diversity found in the Sargasso sea alone as reported by Craig Venter and coworkers[1] identified 1.2 million new genes. Within the *Global Ocean Survey* (GOS) project another 6.1 million new gene sequences were found. As shown by Rusch et al. (2007)[2,3] 1,700 new protein families could be discovered in these databases. This rich source of information are a gold mine for the life sciences as these genes encode for a plethora of novel and mostly unexplored enzymes useful for various areas such as medical science, pharmacy and biocatalysis[4].

During evolution, proteins undergo random mutations that leave their footprint in multiple sequence alignments (MSA). Some amino acid residues will stay conserved, others are conserved in groups of species, and yet others seem to mutate without restrictions. As a result we observe in multiple sequence alignments a hierarchy of residue conservation, correlation, and variation[5–7]. When residues are conserved within groups of sequences that share a certain function but these residues differ between groups we observe correlated mutation behaviour (also called co-evolution), and often such groups of residues are involved in a common function, such as specificity, co-factor binding, protein-protein interactions. We will call such groups of co-evolving residues ‘networks’. CorNet is designed for the analysis of networks and for the prediction of their roles in protein function.

Many attempts have been made to use correlation patterns for the prediction of protein structures using information obtained from a MSA. Older methods all use what is now known as mutual information. A series of CASP[8] experiments illustrated that mutual information obtained from a MSA could not adequately predict protein structures. Recently a series of developments[9–12], have caused a breakthrough in the use of correlated mutations for the *ab initio* prediction of structures. Mutual information has often been related to function[13–16], and distinguishing correlated mutations reflecting residue contacts from those reflecting functions was the major problem faced when predicting protein structure from a MSA. These problems are not encountered, though, when studying or optimizing protein function in fields like protein engineering, chemical biology, or the analysis of disease causing mutations in the human exome because the strongest correlations, and especially whole networks of correlations often reflect a function[14].

Proteins have many functions including ligand and co-factor binding, regulation, signalling, membrane embedding and catalysis. Each function requires that a series of residues work together. Therefore, residues have not co-evolved in a pair-wise manner but rather as networks[17,18]. The concept of extracting correlated mutations from alignments is not new and many methods have been described previously[5,6,13–16,19,20]. Several correlated mutation analysis (CMA) software packages exist (e.g. ET[16], WHAT IF[21]) that cluster detected pairs of residues into networks. Networks are often composed of sub-networks each containing residue positions involved in one particular protein feature. A complicating factor in the assignment of residues to functions is that they often contribute to multiple functions [22,23].

**Fig 1:**
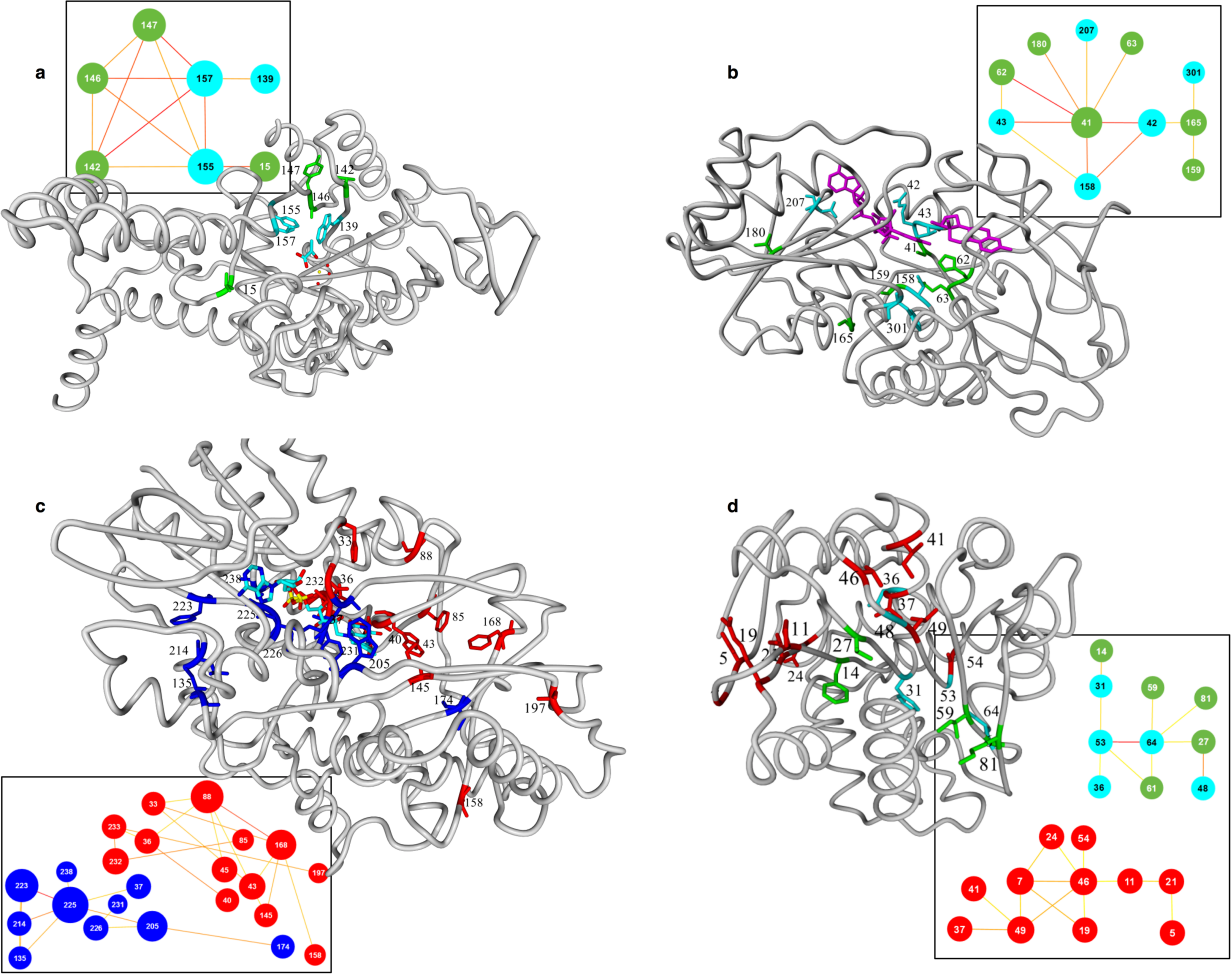
Visualisation of correlated mutation networks in the protein structures. In the boxes the correlated mutation networks are shown. Nodes represent alignment positions. Node sizes indicate the number of edges. Nodes shown in cyan indicate residue positions for which keyword related mutation data is available in the literature. Edge colours indicate the strength of the pair-wise correlation (yellow to red). The residues visualized in the structures correspond with, and match colours with nodes in the network. **a.** Correlated mutation network of the isocitrate lyases (ICL) visualised in structure pdb-code: 1IGW. The cyan nodes in this network are related to the keyword ‘specificity’. **b.** Correlated mutation network of the alcohol dehydrogenases (ADH) visualized in pdb-code: 1D1T that contains a substrate analog in the active site and the NAD co-factor (magenta). Position 41 that is the central hub in the correlation network is also the centre of the 3D network and it is located between the NAD and the substrate-binding pocket. **c.** Correlated mutation network of the amino acid oxidases (AAO) visualized in pdb-code: 1B37 that contains the FAD co-factor. This network consists of two sub-networks (blue surrounding the FAD, red surrounding the substrate binding pocket). **d.** Correlated mutation network of the α/β-hydrolase fold enzymes (a-bH) visualized in pdb-code: 1VA4. This network consists of two sub-networks. The smaller sub-network is highly enriched with positions (cyan nodes) related to the keyword ‘enantioselectivity’.

The function of a network cannot be determined from physicochemical characteristics of the residues involved, but visual inspection of the 3D structure of the protein can reveal the function of a network. Fig 1 shows examples of networks in four super-families that surround ligand- and the cofactor binding pockets. Normally, though, the determination of function requires *in vitro* or *in vivo* experiments, but often such experiments have already been performed in either the molecule of interest or in a homolog and these results can often be extracted from the literature. Besides that the amount of available literature often is overwhelming, a literature study for the functional role of a residue can be complicated by the facts that residues often do not have the same numbers in close homologs and that proteins do not have the same names in different research fields. These problems have been solved in molecular class specific text-mining methods[24,25] that iterate between text analysis and validation using the MSA-based super-family information system.

Six protein super-family systems were used to demonstrate the relation between correlated mutation networks and mutation data that is available in the literature. These six super-families were chosen because they could be made available to the public. We show that very different functions can be the driving force behind the major network in a protein super-family. Specificity is the driving force in two of the six super-families, whereas we observe that twice co-factor binding, once activity, and once enantioselectivity lead to the strongest correlated mutations. Furthermore, We show that randomly deleting a large number of sequences from the input alignment hardly has an effect on the positions that make up the CorNet network. However, deleting entire groups of sequences that are phylogenetically closely related result in CorNet networks consisting of different alignment positions. In fact, when using alignments generated of carefully selected subsets of sequences the networks will reflect different functions compared to networks obtained from the whole super-family. We also show that the enrichment of residues involved in a certain function can be optimised by interactive modification of correlation cut-off values (enrichment is defined as the fraction of residues in the network that is related to the function relative to the fraction of residues related to that function in the whole protein). Enrichment factors between five and ten are not uncommon.

To validate if residues that are connected in a CorNet network indeed share a common function a targeted mutant library was designed for an α/β-hydrolases enzyme (the *Pseudomonas fluorescens* esterase). We experimentally validated the relation between the major network and the associated keyword ‘enantioselectivity’. The analysis of the residue distributions in this network allowed us to design a small library consisting of only 72 variants of which 18% showed a positive effect on enantioselectivity.

The explosion of readily available sequence- and mutation data is likely to make the type of protein data analysis described in this work a standard tool for scientific research in protein engineering and other protein related research fields.

## Results

With the CorNet server the user can select parameters such as correlation scores or colour schemes. The user can rapidly obtain information such as amino acid distributions at single positions or pairs of positions. In the six systems for which we performed the bibliome determination, the user can select search terms in that bibliome and results can be presented as scenes for visualisation of the CorNet data in a protein structure with the Yasara (www.yasara.org) macromolecular structure visualizer (Fig 1 shows examples).

CorNet is connected to the web based CMA tool Comulator and can be used by uploading an alignment to the embedded Comulator tool (www.bio-prodict.nl/comulator). CorNet is also part of the 3DM protein super-family analysis suite. For several publicly available 3DM systems, including the six 3DM databases described in this paper, the alignments, the CMA results, the CorNet networks (including the connection to Yasara), and the mutation data from the bibliome can be retrieved from www.3dm.bio-prodict.nl.

CorNet was tested on six protein super-families: alcohol dehydrogenases (ADH), amino acid oxidase-like (AAO) proteins, RmlC-like cupin proteins (cupins), the phosphoenolpyruvate mutase/isocitrate lyases (ICL), UDP glycosyltransferases (UDP-GT), and α/β-hydrolases (a-bH). We used the Mutator tool[24] to extract from the literature the mutations associated with a series of functions including selectivity, activity, agonist binding, regulation, post-translational modification, and for validation purposes a series of neutral terms such as stability, or the words ‘the’ and ‘and’. In all six families we find that the strongest correlating network clearly relates to a main functional aspect.

### Structural location of correlated mutation networks

Fig 1 shows the structural position of the correlated mutation networks of four superfamilies. Fig 1A and 1B shows the networks for the ICL and ADH superfamilies for which only a single significant network is observed. The AAO and a-bH families reveal a series of significant networks and Fig 1C and 1D show their locations in the respective 3D structures. Fig 1 allows for a series of observations. For example, there is a tendency for residue positions in the same network to also be located roughly in the same area in the 3D structure, but high CMA scores do not tend to relate to 3D contacts. In the AAO family all residues in the blue network are close to the FAD while most residues in the red network are in or near the active site. In none of the six networks do we see that residues that seem central (a hub) in the network are central in their 3D cluster too. The close spatial proximity of network residues seems caused simply by the fact that functions, such as catalysis or co-factor binding, are performed by residues that must lie around the active site or the co-factor. The conclusion that residues in a correlated mutation network will be involved in the same function is corroborated by experimental mutation studies for all of the six super-families studied here. These observations indicate that strongly correlated mutations in multiple sequence alignments are a result of functional constraints rather than structural contacts.

### Residue function determination by enrichment

**Fig 2:**
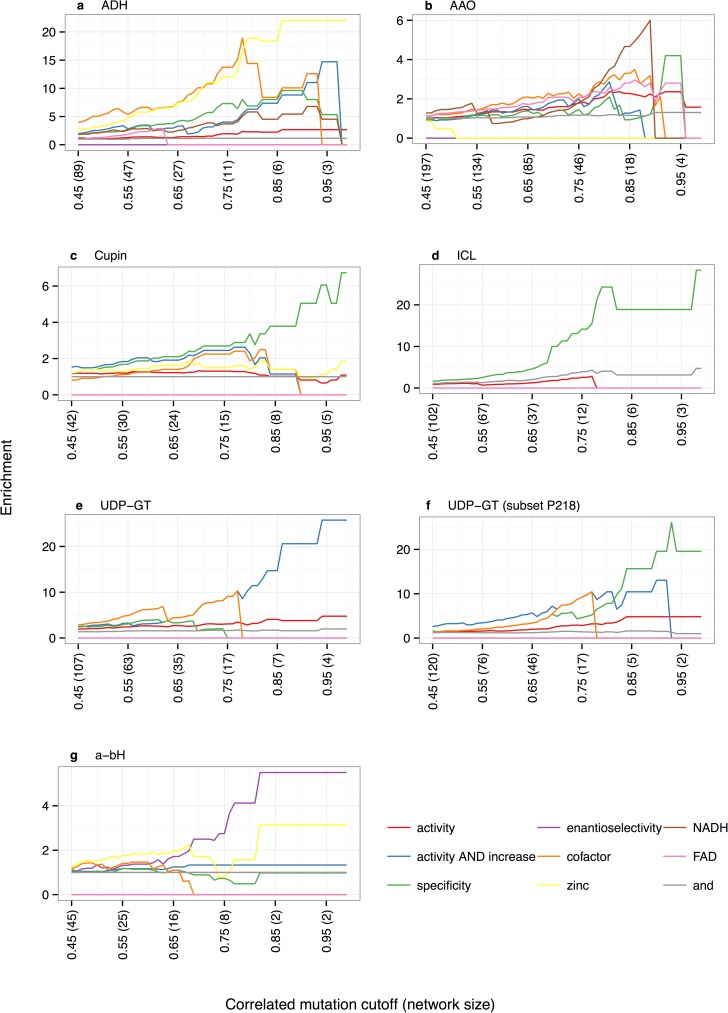
Escores for a series of keywords related to mutations in the families as function of the correlated mutation analysis cut-off. **a.** Keyword enrichments for the alcohol dehydrogenases (ADH). **b.** Keyword enrichments for the Amino acid oxidases (AAO). **c.** Keyword enrichments for the Cupins. **d.** Keyword enrichments for the isocitrate lyases (ICL). **e.** Keyword enrichments for the UDP-Glycosyltransferases (UDP-GT). **f.** Keyword enrichments for a subset of the UDP-Glycosyltransferases (UDP-GT) alignment. This subset is composed of all sequences that have a proline at 3D-number 218. **g.** Keyword enrichments for the α/β-hydrolases (a-bH).

To find the function of residues in a CorNet network literature-extracted mutation data related to different keywords, such as ‘specificity’ and ‘co-factor’, were mapped on the network and the overrepresentation (enrichment) of these keyword-related mutations inside the network is determined by the calculation of enrichment score (Escores). The calculation of Escores is described in the Materials and Methods section. Fig 2 shows for each of these keywords this enrichment in relation to the correlation cut-offs. These enrichments are hard to quantify because of a series of reasons that range from bias in the main research topic in a certain field of the life sciences to low counting statistics caused by, for example, CMA networks reducing to just two amino acid positions at the highest CMA values. Another effect is that researchers tend to make mutations at ‘positions of interest’ and being interesting often is defined by literature describing mutations at that position in homologous proteins. We also observe large differences in the amount of mutation data available per super-family. Originally, we arbitrarily decided that mutations related to a selected keyword had to be observed in at least two independent articles before we would accept it as real. For the ICL and Cupin super-families, this ‘two article’ cut-off had to be abandoned to obtain any results. We do not have enough datasets available yet to start thinking about a relation between the number of available mutation articles, the length of the sequence, the number of sequences in the MSA, and the optimal cut-off for this parameter.

### Enrichment scores

We measured the enrichment for a series of control keywords to at least get a qualitative idea about the significance of Escores. The control keywords ‘and’ and ‘the’ were selected because one expects these words to be observed frequently but randomly in sentences that are picked-up by the logical expressions that scan the literature for sentences that also contain the logical expression for a particular mutation (e.g. P213S). The Escores for these control keywords ranges between 0.00 and 2.02 in five of the super-families (Table 1). We also used the word ‘stability’ and ‘zinc‘ as control keywords. Table 1 shows the enrichments for these four control keywords measured at a CMA value of 0.80. This value was chosen to ensure that the six super-families contain enough nodes to prevent biased enrichment scores, which can result from the fact that scientist tend to select ‘interesting’ positions to mutate. The Network of the ICL super-family is surrounding the active site. Therefore, this biased selection of amino acids results in enriched control keywords simply because there are only a limited number of experimental mutations available.

**Table 1:** Enrichment scores for control keywords^a^.

keyword	and	the	stability	zinc
ADH	1.15	1.18	2.02	–
AAO	1.16	1.15	1.94	0.00
Cupin	1.00	0.94	0.87	1.40
ICL	4.04	3.83	0.00	1.00
UDP-GT	1.54	1.53	0.00	0.00
a-bH	0.00	0.00	1.35	1.57

^**a**^ The enrichments were calculated at a CMA cut-off of 0.80.

The keyword ‘zinc‘ is not shown for the ADH super-family because zinc is a co-factor in this family and thus not a control keyword.

Fig 2 shows for the six super-families the relation between mutations and a series of keywords and their Escores.

#### ADH family

Multiple keywords are enriched for the alcohol dehydrogenase family network (Fig 2A). At a correlation cut-off of 0.80 most of the positions in the network are located in the active site and many of the residues at these positions will likely have more than one function. The difference between the keyword ‘activity’ and the joint-keyword ‘activity AND increase’ should also be noted. Mutations reported in the literature combined with the keyword ‘activity’ are far more evenly distributed over the alignment positions than mutations combined with the keyword ‘activity AND increase’, which is much more enriched for alignment positions within the correlation network. This indicates that many of the positions that can be mutated to increase the activity of these proteins are within the network.

#### AAO family

The amino acid oxidase Escores show that its Network is mainly enriched for ‘FAD’, ‘co-factor’, and ‘specificity’. Fig 1C shows that the AAO network consists of two sub-networks; the one surrounding the FAD cofactor (blue positions) and the other surrounding the substrate-binding pocket (red positions). The enrichments shown in Fig 2B are the sum of the two sub-networks. In fact, mutations related to the keywords ‘FAD’ and ‘cofactor’ are more abundant in the blue sub-network and mutations related to the keyword ‘specificity’ are mostly detected in the red sub-network.

#### Cupin family

Fig 2C shows enrichment for specificity in correlating positions in the cupin super-family Network. At a low cut-off this network shows a low enrichment for ‘activity AND increase’, for ‘co-factor’ and for ‘specificity’. In contrast to the AAO correlation network, the cupin Network is not divided into separate sub-networks. However, a closer investigation of the positions leading to these enrichments revealed that the ‘specificity’ related positions are other positions than the ‘cofactor’ and the ‘activity AND increase’ related positions. S1F Fig shows this network in the 3D structure.

#### ICL family

In the ICL super-family, very high Escores are observed using ‘specificity’ as keyword suggesting that specificity is the driving force causing these residues positions to mutate simultaneously. Inspection of the 3D location of this network reveals that the residues are mainly located in and around the active site (Fig 1A). Escores for the control keyword ‘and’, also illustrated in Fig 2D, show that this keyword is slightly over-represented in this family. Apparently, the majority of the relatively small number of mutations made in proteins of this family are located at positions surrounding the active site probably due to biased selection of residues by scientists.

#### UDP-GT family

The joint-keyword ‘activity AND increased’ is clearly the enriched in the UDP-GT protein super-family network (Fig 2E). Note that, like in the ADH family, the keyword ‘activity’ is hardly enriched in this network. Fig 2F shows that in a subset of the UDP-GT super-family composed by sequences that have a proline at 218, ‘specificity’ clearly has the highest Escore. This subset is discussed in more detail below. S1E Fig shows both the main network and the network for the subset in the 3D structure.

#### a-bH family

In the α/β-hydrolase fold super-family CMA the keyword “enantioselectivity” is clearly enriched (Fig 2G). The Network consists of two sub-networks and most of the mutations effecting enantioselectivity are located in one of the sub-networks (Fig 1D). For five of the ten positions of this sub-network, mutations have been published that effected enantioselectivity (shown in cyan in Fig 1D). To test if the other positions in this network are also important for enantioselectivity a small mutant library was generated for the five non-annotated positions (shown in green in Fig 1D). The positions of the second sub-network cluster spatially, and are lightly enriched for the keyword ‘specificity’.

### Mutant library

The results of an esterase mutation study (Table 2) clearly show the expected impact of the selected correlated network positions on enantioselectivity: 17% of all variants exhibited an improved enantioselectivity (data available in S1 Table) compared to wild-type esterase. Best results were found after the combination of the best mutations obtained at positions 61 (G61S) and 81 (K81H), which led to a 2–3 fold improvement in enantioselectivity.

**Table 2:** Specific activities and apparent enantioselectivity for the top esterase variants.

Variant	Specific activity^a^ [mU/mg]		*E*_app_^b^
	(*R*)-3PB-pNP	(*S*)-3PB-pNP	
*Wild-type*	1.44 (± 0.09)	0.30 (± 0.11)	5
*K81H*	3.22 (± 0.19)	0.54 (± 0.03)	6
*G61S*	4.48 (± 0.72)	0.47 (± 0.04)	10
*G61S/K81H*	6.86 (± 1.08)	0.51 (± 0.03)	13

^a^ One unit corresponds to 1 μmol converted min^-1^ mg^-1^ protein.

^b^ E_app_ is the ratio of activity for the two enantiomer of (*R*)- and (*S*)-3PB-pNP.

A structural analysis of these two positions revealed that position 61 is in the active site region of the esterase from *Pseudomonas fluorescens* (PFE) adjacent to the catalytic aspartic acid, which suggests that a mutation at this position could influence selectivity[26] although the risk is high that catalytic activity can be strongly affected. In contrast position 81 is located on the surface of the protein, far away from the active site. Selection of this position without the CorNet tool and 3DM would have been rather unlikely. The increase in the enantioselectivity is clearly cumulative, although the two positions do not correlate directly to each other in the network.

### Co-evolution networks in alignment subsets

Which function is the underlying force behind a CMA network heavily depends on the input alignment. The Networks of large alignments that cover a large evolutionary spread (e.g. a complete super-family) is composed of different positions compared to a Network of subsets of these alignments that cover only a phylogenetic sub-branch of the large alignment. To investigate the effects of selecting sub-branches on the location of CMA networks in the three-dimensional structure several subgroups of the ADH super-family, the ICL super-family, and the UDP-GT super-family were composed. In all three super-families, sub-alignments were generated by selecting a subgroup of sequences that have a residue conserved at the hub of the main Network.

**Fig 3:**
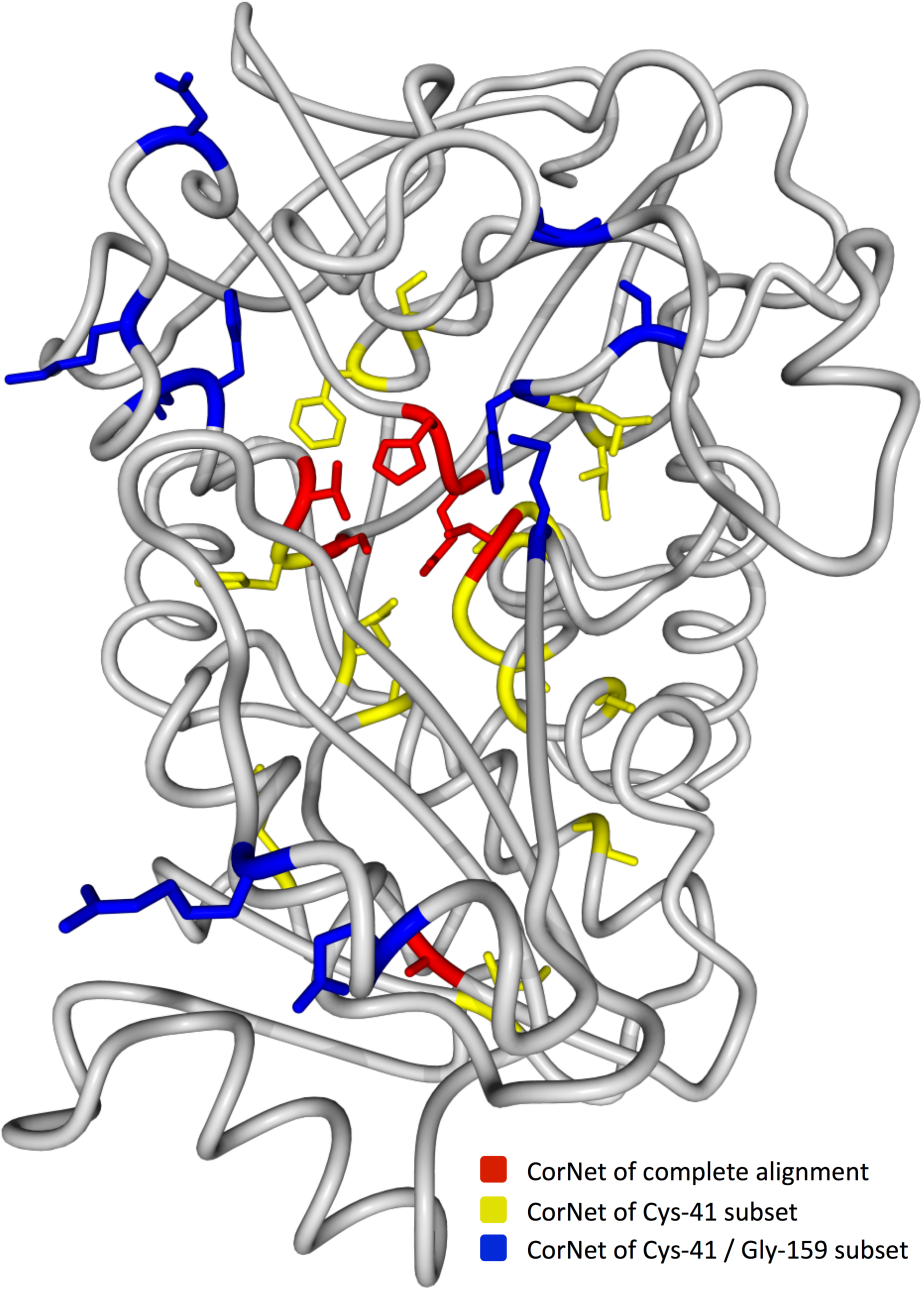
Alcohol dehydrogenase family structure 1CDO-A with CMA network positions of three different alignments visualized. The red residues represent the CMA positions for the complete super-family alignment. The yellow residues represent a network generated for a sub-alignment composed of sequences with a cysteine on 3D-number 41. The blue residues reflect a Network generated for a sub-sub-alignment composed of sequences with a cysteine at position 41 and a glycine at position 159. The catalytic zinc ion is shown in magenta.

The Network of the ADH super-family alignment, for instance, contains a single network and no clear sub-networks can be detected. This network is located in the centre of the active site (red residues Fig 3) and surrounds the zinc ion that is essential for the catalytic activity. Position 41 is a hub in this network (Fig 1B) and makes physical contact with the zinc ion clearly indicated by the high Escore for ‘zinc’ (Fig 2A). The two main residues observed at position 41 are Cys (present in 52.4% of the super-family sequences) and Asn (present in 37.8% of the sequences). A sub-alignment was generated using sequences having a cysteine at alignment position 41. In this subset, position 41 is obviously fully conserved and thus no longer shows up in any correlated mutation network. We observe two new networks in this subset (yellow in Fig 3). These are located surrounding the Network of the complete super-family more in the second layer of the active site. Position 159 is now the main hub in the most extensive network and position 159 mainly occupied by a Gly in the MSA. Using a subset of sequences that have both a Cys at position 41 and a Gly at position 159 we obtain yet another network (blue in Fig 3) positioned in the third layer around the active site. This sub-location of the Networks in different layers around the active site suggests that they reflect different roles (e.g. activity, specificity, dimerization, etc.) that the corresponding residues need to perform. Unfortunately, for the ADH protein family no literature data is yet available that proves this hypothesis and no function could be assigned to the sub-networks with the available literature data.

**Fig 4:**
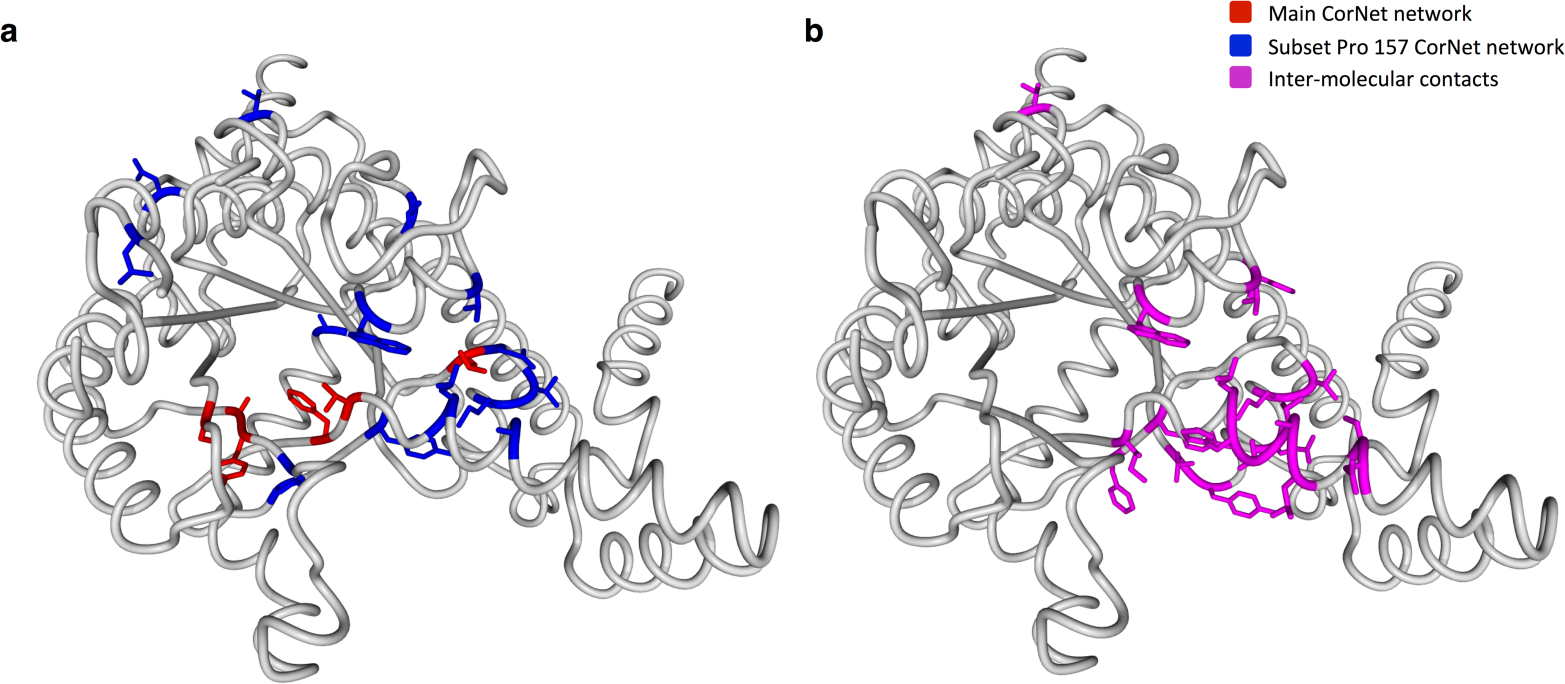
Isocytrate lyases family structure 1DQU-A with CMA networks and dimer interface visualized. **a.** The red residues represent the Network for the complete super-family alignment. The blue residues represent the network for an alignment subset that contains a proline on 3D-number 157. **b.** The purple residues represent the 3D-positions that make an inter-molecular contact in most of the 70 available structures of the ICL family.

The same experiment was performed on the ICL super-family. Position 157 is the main hub of the Network in this super-family and proline is the most common residue at position 157. Fig 2D shows that the main function underlying the Network of this super-family is specificity and this network is located surrounding the substrate-binding pocket (Fig 1A). Although, also for this family, not enough literature-derived mutation data are available to prove the function of the residues in the Network that was generated for a subset containing only sequences with a proline at 157, the network is located almost exclusively at the dimerization interface (Fig 4).

This experiment was repeated in the UPD-GT super-family of which the main network shows a high Escore for the keyword “activity AND increase” (Fig 2E). Position 218 is the main centre of the Network of this super-family and proline is the most common residue at this position. An alignment was generated of all sequences that have a proline at position 218. As shown in Fig 2F in the Network of this alignment the keyword “specificity” results in the highest enrichment.

#### Correlation networks in random subsets

To define the minimal number of sequences needed to perform CMA subsets of randomly selected sequences were generated for all six superfamilies. For each superfamily a range of subsets was generated that contained between 0.5% and 60% of the superfamily sequences. For each subset the network positions were compared to the network of the full alignment and an F-measure was calculated to determine the similarity between the networks. These results (S1 File), show that an alignment of 500 sequences usually contain enough signal to result in a reliable CorNet network indicated by an F-measure of 0.8 or higher.

## Discussion

We describe the protein function—structure—CMA relations for six protein super-families, which were validated using available mutation data from literature. For one of them, the a-bH protein family, a smart mutant library consisting of only 72 variants was designed based on CorNet network to validate the predicted effect on enantioselectivity. To show that positions in a co-evolution network share a common function this library was targeted only at nodes of the network for which no literature mutation data was available describing effects on enantioselectivity. Although the changes observed in enantioselectivity (2–3 fold) are not very large, nearly 20% of the mutants in the library had an effect on enantioselectivity. Typically, random generated libraries have a hit rate of about 1% (Reetz et. al. [27]). This result clearly shows that the positions in a CorNet network are often functionally related. Therefore, mutation information that is available for nodes in a CorNet network can be used to predict the effects of mutating the non-annotated nodes. This experiment was not performed to create a highly enantioselective enzyme (in that case nodes for which effects on enantioselectivity were already published should have been included), but the goal of this experiment was to show that CorNet can be used to find novel mutation hotspots not reported in literature before. In fact, in a recent study we generated a highly selective PFE by mutating a CorNet position for which mutational data was available in the literature [28].

A CMA network, and the function(s) it reflects, depend on the sequences in the alignment. This work shows that not the number of sequences in the alignment, but the evolutionary spread of the aligned sequences is the determining factor for the composition of a CMA network. A large evolutionary spread among the aligned sequences tend to result in a network composed of positions near the active site (i.e. residues performing the main task of the protein). An alignment based on a subset of sequences with a smaller evolutionary spread (i.e. by demanding that one functionally important residue is conserved throughout the subset) results in a correlated mutation network located in the second or third layer of residues (i.e. residues involved in more specific functions). This phenomenon was nicely demonstrated by the difference of enrichments scores in the UDP-GT protein family, where in the full alignment “activity AND increase” resulted in the highest Escore whereas “specificity” scored highest in much smaller subset of the alignment (where P218 is conserved). Rules for determining the best set of input sequences that will result in a Network optimized for a specific protein feature, still remains to be determined. The alignments used in this work were, in fact, automatically generated and no filtering or any form of optimizing was conducted. This shows not only that this method is robust but also that there is still much room for further developments, improvements, and novel discoveries in the area of CMA network related research. The fact that the maximum Escores differ between different super-families and for different protein features suggests that the alignments, and especially the selection of sequences to be included, can be optimized even further. The accuracy of the type of analysis conducted is this work increases when more data is available for a super-family as indicated by high Escores of control keywords in the rather small ICL super-family. Together with the explosion of sequence- and mutation data that is becoming readily available we believe that the type of protein data analysis described in this work might become a standard tool for protein engineering.

## Materials and methods

### Protein families

The relation between correlation networks and information from the bibliome was analysed for six super-families. *ADH*: Alcohol dehydrogenases catalyse the oxidation of alcohols by the reduction of nicotinamide adenine dinucleotide. *AAO*: Amino acid oxidases are FAD-binding proteins. This family consists of two sub-families that catalyse *D*-amino acid and *L*-amino acid conversion, respectively. These two AAO sub-families bind their FAD differently. *Cupin*: The very large RmlC-like cupin family comprises a wide range of enzymes that can convert many different substrates. Cupins show a large variety of reaction mechanisms. The cupins are the most diverse protein family known today covering 17 enzyme classes and even other types of proteins such as seed storage globulins and multi-domain transcription factors[29]. *ICL*: The phosphoenolpyruvate mutase/isocitrate lyases super-family contains several enzyme families that act on alpha-oxycarboxylate substrates. *UDP-GT*: The UDP-Glycosyltransferases protein family contains sugar-acting enzymes that can act on different sugars and perform different reactions (synthases, transferases, phosphorylases). *a-bH*: The α/β-hydrolase fold super-family contains a wide range of proteins including proteases, esterases and lipases[30].

For each of these six families structure based MSAs were produced, and the literature was scanned for mutations. Table 3 lists the number of articles, sequences, structures, core alignment positions, and mutations found for each of the six protein super-families.

**Table 3:** Sequences, structures, and mutations found for the six super-families.

Name	Sequences	Core alignment positions	Structures	Articles scanned	Mutation data extracted
ADH	14696	353	447	15144	10437
AAO	12155	253	356	14442	6203
Cupin	1650	43	338	53400	4362
ICL	3019	170	70	2013	160
UDP-GT	36402	313	475	26919	7610
a-bH	59904	88	1665	60926	60755

The CMA scores were determined for all pairs of alignment positions in each of the six families. Mutual information was calculated rather than the direct information that has been described[9–12]. Correlation scores are obtained using the previously described Comulator software[14]. Comulator uses a method known as a statistical coupling analysis[31,32] to assign correlation scores. Comulator was used because this method is a robust CMA algorithm that was specifically developed to handle large structure based superfamily alignments that consist of thousands of proteins and often contain many different protein functions.

### Cornet features

The CMA network visualization tool was built using cytoscape.js (a JavaScript graph visualization library)[33] and jquery (user interface libraries). In this HTML based network viewer nodes represent alignment positions (with the MSA position number indicated) and edges are coloured as function of the pairwise CMA values. The nodes are hyperlinked to underlying data stored in the database so that, for example, the amino acid distribution of an alignment position or a pair of correlating positions can be obtained rapidly.

The user can interactively choose correlation cut-offs, colours for groups of residue positions, and residue positions can be coloured as function of their annotation. CorNet can write the resulting colours in a YASARA scene so that results can be visualised with YASARA, a protein structure visualisation tool (Fig 1). When annotation queries are performed, the enrichment of the search term is determined on the fly.

### Mutation extraction

The Mutator software[24] was used for the extraction of mutations from the literature. This software searches in PubMed with words like ‘mutation’, ‘SNP’ (Single Nucleotide Polymorphism), ‘substitution’, or ‘recombinant' combined with family-specific keywords (and their synonyms) like names of family members, their gene names, names of diseases known to be related to members of the family, or generic family names. Most names are retrieved from the Swiss-Prot[34] protein entries available in the MSA. We subsequently scan these articles for mutation information related to the six super-families. Each sentence that contained a mutation (i.e. terms like S127P, Glu422Lys or “Trp58 was mutated to Ala”) was analysed for the presence of a series of words such as ‘specificity’, ‘activity’, ‘cofactor’, etc. A residue position is considered related to a keyword if the combination of mutation and keyword is observed in the same sentence in at least two articles that describe a mutation at the same residue position in a member of the family.

### 3DM

3DM was used to generate the structure bases multiple sequence alignments (MSAs) for the six super-families[35]. In summary, structures are superposed with WHAT IF [21] to generate an initial alignment that is then used to guide the alignment of all sequences for which no structure data is available. 3DM allows for the generation of alignments for subgroups of sequences. Such sub-alignments were generated for the UPD- and ADH protein families. These sub-alignments are composed of all sequences that have the most abundant residue at the position that forms the centre of the Network. Correlated mutation analysis is performed as described before[14]. A method known as statistical coupling analysis[32] is used to detect pairs of residues in the alignment that mutate simultaneously.

### 3D numbers

**Fig 5:**
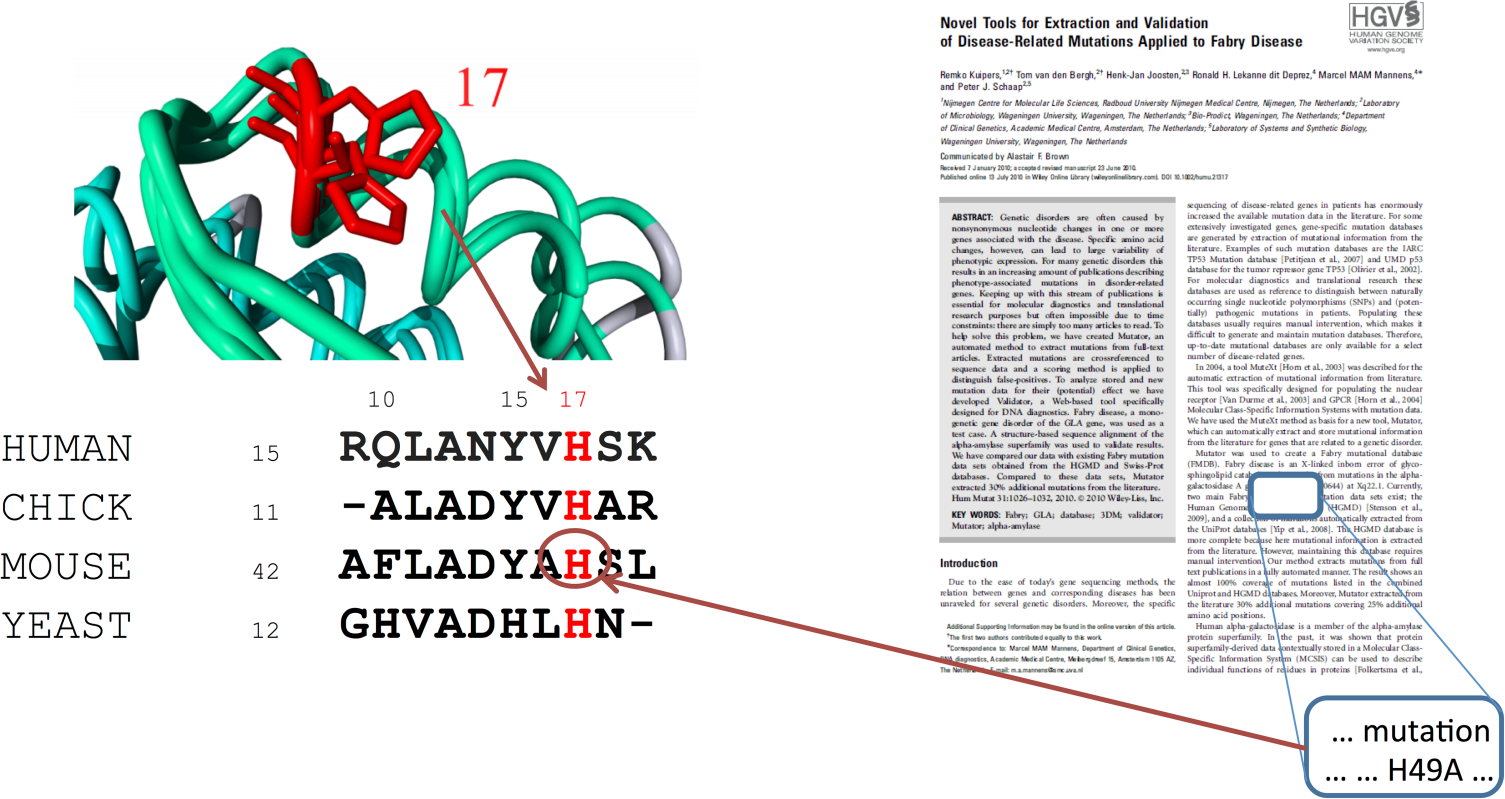
Example to illustrate the use of 3D-numbers. We are interested in histidine 22 in the human sequence, however mutation related information from the bibliome is only available for the mouse homologous sequence. In the main text we find a description of the effect of a mutation of histidine 49 to an alanine. This histidine residue is in the structure at equivalent position of the human histidine-22 and therefore shares the same 3D number (17).

CorNet uses a super-family specific residue numbering scheme for all sequences and structures in the alignment. Structurally equivalent residues get the same numbers, called 3D-numbers, which are also used for the corresponding sequence alignment positions. 3D-numbering schemes are used throughout this paper, and in the interactive version of CorNet. The principle of 3D- numbers and the underlying structure based multiple sequence alignment have been described[35] and is illustrated in Fig 5. Structurally variable sites such as residues that reside in loops are not included in the structure based MSA and thus are not included in the correlation analyses. In practice, though, functionally important residues normally are located in the structurally conserved regions of proteins.

### Escore and P-values

The keyword search option enables the user to automatically select mutations for which that keyword is part of the annotation and to map these on the network. The overrepresentation of a keyword for residues in a network is expressed as the enrichment-score (Escore)_:_

$$Escore=(K_{n}/K_{t})*(N_{t}/N_{n})$$

**Equation 1**. N_n_ = number of alignment positions in the network, N_t_ = total number of alignment positions, K_n_ = number of network positions for which the keyword m was observed, K_t_ = total number of positions for which keyword m was observed.

CorNet offers the user to define a cut-off (N_mut_)_,_ which can be selected interactively; the default is 2. The keyword must be observed with the same 3D residue position in at least N_mut_ mutation studies for different proteins in order to be accepted.

### Library design

The design of the mutant library composed of 72 variants was based on a 3DM analysis of the respective positions, which led to the incorporation of the four most frequent amino acids at the networks positions (Table 4): a triple mutant library was designed to include the combinatorial effects of those positions that either are connected with more than one node with a known effect on enantioselectivity (i.e. nodes 27 and 61, Fig 1D) or with nodes that have been more frequently mutated according to literature (i.e. node 14, Fig 1D). The remaining two positions (i.e. nodes 59 and 81) were randomized independently.

**Table 4:** 3D positions selected, codons used for library design and corresponding encoded amino acids.

3D position	Codons	Amino acids encoded
*14*	TKG/TWT	L,W,**F**,Y
*27*	GBC/ACC	V,A,G,**T**
*59*	VTT/GGT	V,**I**,L,G
*61*	GSC/ARC	**G**,A,N,S
*81*	YAT/CGT/GTT	H,Y,R,V

Residues in bold correspond to wild-type esterase.

#### Mutant libraries and enantioselectivity

Libraries of the esterase from *Pseudomonas fluorescens* (PFE) were constructed by QuikChange mutagenesis. In the case of the triple mutant library three consecutive reactions were needed. In each case the following reaction mixture was prepared: sterilized deionized H_2_O (41 μL), Pfu buffer (10x, 5 μL), dNTP (1 μL, 10 mM each), plasmid pJOE2792.1 (1 μL, 50 nmol μL^-1^) containing the gene encoding PFE[36], mixture of forward and reverse primer mixture (1 μL, 12.5 nmol μL^-1^), Pfu^+^ DNA Polymerase (0.2 μL). The so prepared mixture was then split in two different PCR tubes with equal amount of volumes and used for a PCR at the following conditions: 1) 95°C, 300 s; 2) 30 cycles: 95°C, 30 s; 50 or 65°C, 30 s; 72°C 210 s; 3) 72°C, 480 s. Afterwards, the presence of the PCR product was verified on a 1% agarose gel and finally DpnI (0.5 μl) was added to remove the template. Digestion of the most abundant product was performed for 2 h at 37°C followed by denaturation of DpnI at 80°C for 20 minutes. Chemo-competent *E*. *coli* cells (Top10) were transformed with the PCR product for plasmid amplification and quality library evaluation[37]. Once the randomization state of the mutated position was verified by sequencing, the mixture of circularized plasmids was used for transformation in chemo-competent *E*.*coli* cells (BL21 DE3) and plated onto LB_AMP_-plates. Clones were picked with sufficient oversampling (3-fold) to ensure statistically a 95% coverage of the library[38].

### Primers:

**1afw—**5’-GGTGTTGTKGAGCCACGGTTGGCTACTGG-3’,
**1bfw—**5’-GGTGTTGTWTAGCCACGGTTGGCTACTGG-3’,
**1arv—**5’-CGTGGCTCMACAACACCGGTTTACCGCTGC-3’,
**1brv—**5’CGTGGCTAWACAACACCGGTTTACCGCTGC-3’,
**2afw—**5’-CCTCAAGGAGGTGGBCCTGGTGGGCTTCTCC-3’,
**2bfw—**5’-CCTCAAGGAGGTGACCCTGGTGGGCTTCTCC-3’,
**2arv—**5’-GGAGAAGCCCACCAGGVCCACCTCCTTGAGG-3’,
**2brv—**5’-GGAGAAGCCCACCAGGGTCACCTCCTTGAGG-3’,
**3afw—**5’- CCACCCTGGTGVTTCATGGCGATGG-3’,
**3bfw—**5’- CCACCCTGGTGGGTCATGGCGATGG-3’,
**3arv—**5’-CCATCGCCATGAABCACCAGGGTGG-3’,
**3brv—**5’- CCATCGCCATGACCCACCAGGGTGG-3’,
**4afw—**5’-GTGATCCATGSCGATGGCGACC-3’,
**4bfw—**5’- GTGATCCATARCGATGGCGACC-3’,
**4arv—**5’- GGTCGCCATCGSCATGGATCAC-3’,
**4brv—**5’- GGTCGCCATCGYTATGGATCAC-3’,
**5afw—**5’- CGAACTGYATGTGTACAAGGACG-3’,
**5bfw—**5’- CGAACTGCGTGTGTACAAGGACG:-3’,
**5cfw—**5’- CGAACTGGTTGTGTACAAGGACG-3’,
**5arv—**5’- CGTCCTTGTACACATRCAGTTCG-3’,
**5brv—**5’- CGTCCTTGTACACACGCAGTTCG-3’,
**5crv—**5’- CGTCCTTGTACACAACCAGTTCG-3’,
**6afw—**5’- GCCGAACTGCATGTGTACAAGGACGCGCCCCACG-3’,
**6arv—**5’- CCTTGTACACATGCAGTTCGGCGCCCTTGATCAAC-3’,
**7afw—**5’- GGTGGTGCATAGCGATGGCGACCAGATCG-3’**,**
**8arv—**5’- CGCTATGCACCACCAGGGTGGGTACGTC-3’.

The primers series **1**, **2** and **4** were used for the randomization of positions 14, 27 and 61, respectively, in the triple mutant library. Primers series **3** and **5** were used for the independent randomizations at positions 59 and 81 respectively. Primers series **6** and **7** were used for the creation of the single mutants derived from the combination of the best hits at each network node.

For protein expression, the transformants were grown on agar plates, picked and inoculated into microtiter plates containing 200 μL LB_AMP_. Incubation was performed overnight at 37°C and 500 rpm. The following day the overnight culture (50 μL) was transferred into deep-well blocks containing 1 mL TB_AMP_ and incubated for 3 h at 37°C at 700 rpm. Gene expression was induced with L-rhamnose solution (final concentration 0.2% (w/v)). The libraries were incubated for an additional 16 h at 30°C, 700 rpm. For disruption, cells were harvested by centrifugation (15 min, 4355 g and 4°C) and resuspended in 300 μL lysis buffer containing 1% Bugbuster solution for 1 h at 37°C at 700 rpm followed by centrifugation for 45 min at 4355 g, 4°C. The crude cell extract was transferred into a new microtiter plate and stored until usage at 4°C. For each variant the crude cell lysate was split into two microtiter plates containing phosphate buffer (50 mM, pH 7.5). Enantioselectivity measurements were performed in microtiter plates (MTP) first with crude cell lysate using optically pure (*R*)- and (*S*)-3-phenylbutyric acid-*p*-nitrophenylesters (0.2 mM final concentration in 20% acetonitrile, synthesized as described previously[39]) in two separate wells of the MTP for each variant following for 1 h the increase in absorbance at 410 nm from the released *p*-nitrophenolate. From the difference in the rate of the hydrolysis of the two enantiomers, the apparent enantioselectivity was determined as described previously[39]. Variants showing improved properties in this initial screening were produced on larger scale, His-tag purified using TALON beads and reanalyzed for altered enantioselectivity.

## Supporting information

S1 Table Complete list of the screened mutants with respective ‘BLANK’-wells (no enzyme) and ‘wt’-wells (containing wild-type enzyme). (DOCX)

S1 Fig Visualisation of correlated mutation networks in the protein structures. In the boxes the correlated mutation networks are shown. Nodes represent alignment positions. Node sizes indicate the number of edges. Nodes shown in cyan indicate residue positions for which keyword related mutation data is available in the literature. Edge colours indicate the strength of the pair-wise correlation (yellow to red). The residues visualized in the structures correspond with, and match colours with nodes in the network. **a.** Correlated mutation network of the isocitrate lyases (ICL) visualised in structure pdb-code: 1IGW. The cyan nodes in this network are related to the keyword ‘specificity’. **b.** Correlated mutation network of the alcohol dehydrogenases (ADH) visualized in pdb-code: 1D1T that contains a substrate analog in the active site and the NAD co-factor. Position 41 that is the central hub in the correlation network is also the centre of the 3D network and it is located between the NAD and the substrate-binding pocket. **c.** Correlated mutation network of the amino acid oxidases (AAO) visualized in pdb-code: 1B37 that contains the FAD co-factor. This network consists of two sub-networks (blue surrounding the FAD, red surrounding the substrate binding pocket). **d.** Correlated mutation network of the α/β-hydrolase fold enzymes (a-bH) visualized in pdb-code: 1VA4. This network consists of two sub-networks. The smaller sub-network is highly enriched with positions (cyan nodes) related to the keyword ‘enantioselectivity’. **e.** Correlated mutation network of the UDP-Glycosyltransferases (UDP-GT) visualized in pdb-code: 3S28. The red network results from the full alignment. The blue network results from a subset of the alignment. This subset is composed of all sequences that have a proline at 3D-number 218. **f.** Correlated mutation network of the Cupins visualized in pdb-code: 1CAU.
(TIFF)

S1 File File containing accuracy and F-measure scores for the different samples of sequences selected from the alignments. (XLSX)
