# Supplementary material for: CorNet: Assigning function to networks of co-evolving residues by automated literature mining
Source: PLoS One. 2017 May 18;12(5):e0176427. doi: 10.1371/journal.pone.0176427 (PMC5436653; doi:10.1371/journal.pone.0176427)
Supplement: S1 Table — (DOCX) [file pone.0176427.s001.docx]

**Table S1.** Complete list of the screened mutants with respective ‘BLANK’-wells (no enzyme) and ‘wt’-wells (containing wild-type enzyme).

| well type | **Plate 1** | **Eapp^[a]^** | well type | **Plate 2** | **Eapp^[a]^** | well type | **Plate 3** | **Eapp^[a]^** | well type | **Plate 4** | **Eapp^[a]^** |
| --- | --- | --- | --- | --- | --- | --- | --- | --- | --- | --- | --- |
| **mutants**  **library** | A01 | 0.84 | **mutants**  **library** | A01 | 1.07 | **mutants**  **library** | A01 | 1.13^[d]^ | **mutants**  **library** | A01 | 0.98 |
|  | A02 | 4.36^[b]^ |  | A02 | 1.02 |  | A02 | 1.12 |  | A02 | 1.07 |
|  | A03 | 0.82 |  | A03 | 1.65 |  | A03 | 2.44 |  | A03 | 3.55 |
|  | A04 | 0.94^[c]^ |  | A04 | 1.01 |  | A04 | 1.53 |  | A04 | 1.08 |
|  | A05 | 0.85 |  | A05 | 1.83 |  | A05 | 1.45 |  | A05 | 1.16 |
|  | A06 | 0.87 |  | A06 | 0.97 |  | A06 | 2.67 |  | A06 | 1.02 |
|  | A07 | 0.83 |  | A07 | 1.07 |  | A07 | 2.15 |  | A07 | 1.08 |
|  | A08 | 0.90 |  | A08 | 1.04 |  | A08 | 1.08 |  | A08 | 1.00 |
|  | A09 | 0.86 |  | A09 | 3.88 |  | A09 | 1.45 |  | A09 | 0.94 |
|  | A10 | 0.83 |  | A10 | 1.02 |  | A10 | 1.03 |  | A10 | 1.02 |
|  | A11 | 0.81 |  | A11 | 0.91 |  | A11 | 2.62 |  | A11 | 1.09 |
|  | A12 | 0.90 |  | A12 | 1.09 |  | A12 | 1.01 |  | A12 | 0.85 |
|  | B01 | 3.08 |  | B01 | 1.05 |  | B01 | 1.25 |  | B01 | 5.33 |
|  | B03 | 0.78^[e]^ |  | B03 | 0.98 |  | B03 | 1.14 |  | B03 | 1.11 |
|  | B04 | 0.83 |  | B04 | 3.46 |  | B04 | 1.08 |  | B04 | 5.04 |
|  | B05 | 0.85 |  | B05 | 0.96 |  | B05 | 1.19 |  | B05 | 4.53 |
|  | B06 | 0.80 |  | B06 | 0.94 |  | B06 | 2.63 |  | B06 | 1.08 |
|  | B07 | 0.87 |  | B07 | 0.97 |  | B07 | 1.16 |  | B07 | 1.07 |
|  | B08 | 0.84 |  | B08 | 0.97 |  | B08 | 1.11 |  | B08 | 0.96 |
|  | B09 | 0.80 |  | B09 | 1.03 |  | B09 | 1.15 |  | B09 | 0.94 |
|  | B10 | 0.84 |  | B10 | 0.90 |  | B10 | 2.70 |  | B10 | 1.31 |
|  | B12 | 0.90 |  | B12 | 1.07 |  | B12 | 1.12 |  | B12 | 1.14 |
|  | C01 | 6.03 |  | C01 | 0.98 |  | C01 | 1.19 |  | C01 | 0.97 |
|  | C02 | 0.82 |  | C02 | 0.96 |  | C02 | 1.16 |  | C02 | 1.00 |
|  | C03 | 0.78 |  | C03 | 1.23 |  | C03 | 1.37 |  | C03 | 1.12 |
|  | C04 | 4.56 |  | C04 | 0.99 |  | C04 | 1.06 |  | C04 | 1.13 |
|  | C05 | 0.82 |  | C05 | 0.95 |  | C05 | 1.16 |  | C05 | 0.99 |
|  | C06 | 0.83 |  | C06 | 1.00 |  | C06 | 1.13 |  | C06 | 1.58 |
|  | C07 | 0.80 |  | C07 | 2.62 |  | C07 | 1.12 |  | C07 | 1.09 |
|  | C08 | 0.82 |  | C08 | 0.99 |  | C08 | 1.49 |  | C08 | 0.99 |
|  | C09 | 2.93 |  | C09 | 3.87 |  | C09 | 1.05 |  | C09 | 0.87 |
|  | C10 | 2.32 |  | C10 | 1.00 |  | C10 | 1.02 |  | C10 | 1.01 |
|  | C11 | 0.73 |  | C11 | 1.02 |  | C11 | 1.08 |  | C11 | 2.87 |
|  | C12 | 0.94 |  | C12 | 1.04 |  | C12 | 1.15 |  | C12 | 2.18 |
|  | D01 | 0.85 |  | D01 | 0.97 |  | D01 | 1.09 |  | D01 | 2.63 |
|  | D02 | 5.69 |  | D02 | 0.97 |  | D02 | 1.12 |  | D02 | 2.53 |
|  | D03 | 0.79 |  | D03 | 4.78 |  | D03 | 1.09 |  | D03 | 2.69 |
|  | D04 | 0.83 |  | D04 | 1.06 |  | D04 | 2.85 |  | D04 | 2.89 |
|  | D05 | 0.90 |  | D05 | 3.99 |  | D05 | 1.12 |  | D05 | 2.71 |
|  | D06 | 0.79 |  | D06 | 0.99 |  | D06 | 1.30 |  | D06 | 2.72 |
|  | D07 | 0.80 |  | D07 | 0.99 |  | D07 | 1.04 |  | D07 | 3.10 |
|  | D08 | 0.84 |  | D08 | 1.03 |  | D08 | 1.12 |  | D08 | 3.12 |
|  | D09 | 1.12 |  | D09 | 1.19 |  | D09 | 2.94 |  | D09 | 2.71 |
|  | D10 | 0.82 |  | D10 | 1.01 |  | D10 | 1.07 |  | D10 | 2.79 |
|  | D11 | 0.86 |  | D11 | 0.91 |  | D11 | 1.08 |  | D11 | 2.85 |
|  | D12 | 0.84 |  | D12 | 1.05 |  | D12 | 1.10 |  | D12 | 2.50 |
|  | E01 | 0.84 |  | E01 | 1.02 |  | E01 | 2.24 |  | E01 | 2.47 |
|  | E02 | 0.83 |  | E02 | 0.93 |  | E02 | 1.13 |  | E02 | 2.78 |
|  | E03 | 0.80 |  | E03 | 1.19 |  | E03 | 2.87 |  | E03 | 2.73 |
|  | E04 | 0.83 |  | E04 | 0.97 |  | E04 | 1.01 |  | E04 | 2.58 |
|  | E05 | 0.91 |  | E05 | 2.03 |  | E05 | 1.12 |  | E05 | 2.69 |
|  | E06 | 0.81 |  | E06 | 1.02 |  | E06 | 2.53 |  | E06 | 2.53 |
|  | E07 | 0.83 |  | E07 | 1.00 |  | E07 | 1.11 |  | E07 | 2.74 |
|  | E08 | 4.63 |  | E08 | 0.96 |  | E08 | 1.20 |  | E08 | 3.02 |
|  | E09 | 0.79 |  | E09 | 1.49 |  | E09 | 1.11 |  | E09 | 1.68 |
|  | E10 | 4.68 |  | E10 | 1.01 |  | E10 | 1.11 |  | E10 | 3.13 |
|  | E11 | 0.78 |  | E11 | 0.93 |  | E11 | 2.37 |  | E11 | 2.92 |
|  | E12 | 0.83 |  | E12 | 1.03 |  | E12 | 1.07 |  | E12 | 2.74 |
|  | F01 | 0.84 |  | F01 | 1.09 |  | F01 | 1.11 |  | F01 | 2.94 |
|  | F02 | 0.82 |  | F02 | 1.07 |  | F02 | 1.11 |  | F02 | 3.06 |
|  | F03 | 0.80 |  | F03 | 0.99 |  | F03 | 1.08 |  | F03 | 2.51 |
|  | F04 | 0.83 |  | F04 | 2.76 |  | F04 | 1.08 |  | F04 | 2.65 |
|  | F05 | 0.89 |  | F05 | 1.02 |  | F05 | 2.20 |  | F05 | 2.68 |
|  | F06 | 0.78 |  | F06 | 2.82 |  | F06 | 1.12 |  | F06 | 3.00 |
|  | F07 | 1.06 |  | F07 | 2.87 |  | F07 | 1.12 |  | F07 | 2.70 |
|  | F08 | 0.78 |  | F08 | 1.01 |  | F08 | 1.07 |  | F08 | 1.06 |
|  | F09 | 0.98 |  | F09 | 1.07 |  | F09 | 1.39 |  | F09 | 1.11 |
|  | F10 | 0.77 |  | F10 | 0.96 |  | F10 | 0.81 |  | F10 | 2.67 |
|  | F11 | 0.82 |  | F11 | 0.96 |  | F11 | 1.02 |  | F11 | 1.10 |
|  | F12 | 5.34 |  | F12 | 1.17 |  | F12 | 2.95 |  | F12 | 2.71 |
|  | G01 | 0.82 |  | G01 | 0.93 |  | G01 | 1.13 |  | G01 | 1.03 |
|  | G03 | 4.46 |  | G02 | 1.00 |  | G02 | 1.12 |  | G02 | 1.06 |
|  | G04 | 0.86 |  | G04 | 0.99 |  | G03 | 2.06 |  | G03 | 1.98 |
|  | G05 | 0.87 |  | G05 | 1.28 |  | G05 | 1.12 |  | G04 | 1.05 |
|  | G06 | 0.79 |  | G06 | 0.95 |  | G06 | 1.06 |  | G06 | 2.56 |
|  | G07 | 0.81 |  | G07 | 0.96 |  | G07 | 1.10 |  | G07 | 2.32 |
|  | G08 | 0.84 |  | G08 | 0.96 |  | G08 | 1.06 |  | G09 | 1.06 |
|  | G09 | 0.84 |  | G09 | 1.00 |  | G10 | 3.27 |  | G10 | 1.03 |
|  | G10 | 0.77 |  | G11 | 2.05 |  | G11 | 1.17 |  | G11 | 1.56 |
|  | G12 | 0.87 |  | G12 | 1.01 |  | G12 | 1.10 |  | G12 | 2.54 |
| **BLANK** | B02 | 0.83 | **BLANK** | B02 | 1.36 | **BLANK** | B02 | 1.18 | **BLANK** | B02 | 1.18 |
|  | B11 | 0.84 |  | B11 | 0.82 |  | B11 | 1.24 |  | B11 | 0.96 |
|  | G02 | 0.80 |  | G03 | 1.04 |  | G04 | 1.14 |  | G05 | 1.02 |
|  | G11 | 0.79 |  | G10 | 1.01 |  | G09 | 1.52 |  | G08 | 1.20 |
| **wt** | H01 | 2.55 | **wt** | H01 | 2.50 | **wt** | H01 | 2.35 | **wt** | H01 | 2.58 |
|  | H02 | 2.55 |  | H02 | 2.66 |  | H02 | 2.58 |  | H02 | 2.61 |
|  | H03 | 2.56 |  | H03 | 2.36 |  | H03 | 2.50 |  | H03 | 2.64 |
|  | H04 | 2.62 |  | H04 | 2.31 |  | H04 | 2.56 |  | H04 | 2.75 |
|  | H05 | 2.56 |  | H05 | 2.47 |  | H05 | 2.69 |  | H05 | 2.39 |
|  | H06 | 2.45 |  | H06 | 2.42 |  | H06 | 2.40 |  | H06 | 2.63 |
|  | H07 | 2.67 |  | H07 | 2.31 |  | H07 | 2.64 |  | H07 | 2.58 |
|  | H08 | 2.43 |  | H08 | 2.36 |  | H08 | 2.61 |  | H08 | 2.71 |
|  | H09 | 2.45 |  | H09 | 2.56 |  | H09 | 2.70 |  | H09 | 2.58 |
|  | H10 | 2.38 |  | H10 | 2.32 |  | H10 | 2.51 |  | H10 | 2.48 |
|  | H11 | 2.70 |  | H11 | 2.48 |  | H11 | 2.61 |  | H11 | 2.36 |
|  | H12 | 2.64 |  | H12 | 2.48 |  | H12 | 2.76 |  | H12 | 2.69 |
| [a] The Eapp was calculated as the ratio of activities; (ΔOD/min)_(_*_R_*_)_/(ΔOD/min)_(_*_S_*_)_ [b] Indicates that E_app_HIT was higher than the highest E_app_wt (out of 12 replicates per plate). [c] Indicates that the E_app_HIT was higher than the highest E_app_BLANK (out of 4 replicates per plate and equal to the compound’s auto-hydrolysis in aqueous solution) and lower than the lowest E_app_wt (out of 12 replicates per plate). [d] Indicates that the E_app_HIT was lower than the lowest E_app_BLANK (out of 4 replicates per plate and equal to the compound’s auto-hydrolysis in aqueous solution). [e] Indicates that the E_app_HIT was lower than 1 and lower than the lowest E_app_BLANK (out of 4 replicates per plate and equal to the compound’s auto-hydrolysis in aqueous solution). | | | | | | | | | | | |
