# Supplementary figures and images for: CorNet: Assigning function to networks of co-evolving residues by automated literature mining
Source: PLoS One. 2017 May 18;12(5):e0176427. doi: 10.1371/journal.pone.0176427 (PMC5436653; doi:10.1371/journal.pone.0176427)

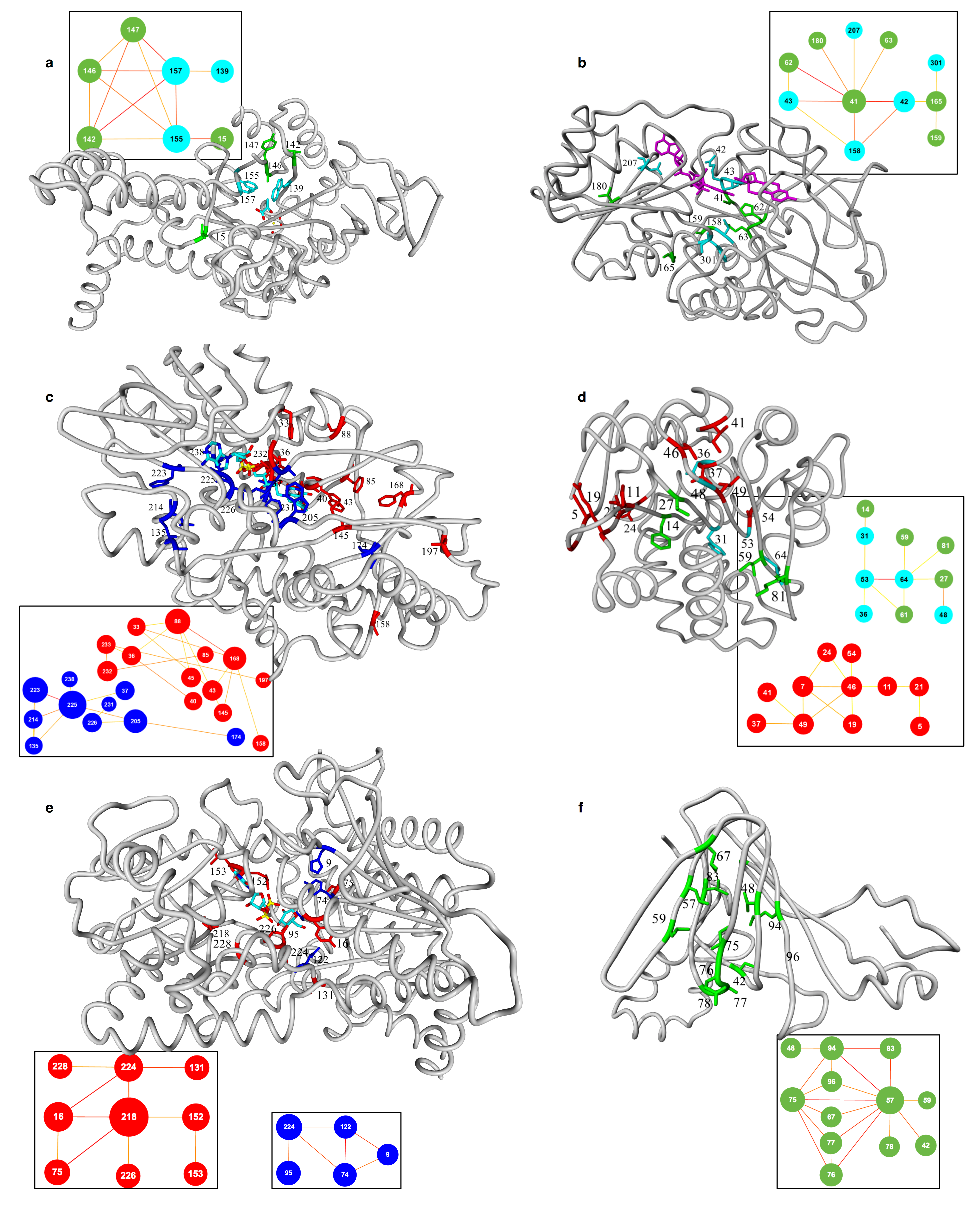

Supplement: S1 Fig — In the boxes the correlated mutation networks are shown. Nodes represent alignment positions. Node sizes indicate the number of edges. Nodes shown in cyan indicate residue positions for which keyword related mutation data is available in the literature. Edge colours indicate the strength of the pair-wise correlation (yellow to red). The residues visualized in the structures correspond with, and match colours with nodes in the network. a. Correlated mutation network of the isocitrate lyases (ICL) visualised in structure pdb-code: 1IGW. The cyan nodes in this network are related to the keyword ‘specificity’. b. Correlated mutation network of the alcohol dehydrogenases (ADH) visualized in pdb-code: 1D1T that contains a substrate analog in the active site and the NAD co-factor. Position 41 that is the central hub in the correlation network is also the centre of the 3D network and it is located between the NAD and the substrate-binding pocket. c. Correlated mutation network of the amino acid oxidases (AAO) visualized in pdb-code: 1B37 that contains the FAD co-factor. This network consists of two sub-networks (blue surrounding the FAD, red surrounding the substrate binding pocket). d. Correlated mutation network of the α/β-hydrolase fold enzymes (a-bH) visualized in pdb-code: 1VA4. This network consists of two sub-networks. The smaller sub-network is highly enriched with positions (cyan nodes) related to the keyword ‘enantioselectivity’. e. Correlated mutation network of the UDP-Glycosyltransferases (UDP-GT) visualized in pdb-code: 3S28. The red network results from the full alignment. The blue network results from a subset of the alignment. This subset is composed of all sequences that have a proline at 3D-number 218. f. Correlated mutation network of the Cupins visualized in pdb-code: 1CAU. (TIFF) [file pone.0176427.s002.tiff]
